# Noninvasive measurement of blood calcium concentration using electrocardiography in peripartum Jersey cows

**DOI:** 10.3389/fvets.2023.1198367

**Published:** 2023-10-11

**Authors:** Yuto Chiba, Isamu Nozaki, Megumi Itoh, Satoshi Kawamoto

**Affiliations:** ^1^Department of Veterinary Medicine, Obihiro University of Agriculture and Veterinary Medicine, Obihiro, Japan; ^2^School of Veterinary Medicine, Rakuno Gakuen University, Ebetsu, Japan

**Keywords:** calving number, corrected ST interval, estimation, hypocalcemia, regression equation

## Abstract

We previously developed a noninvasive method for measuring blood calcium concentration (Ca) in Holstein cows on site using electrocardiographic (ECG) variables and calving number, based on a high positive correlation between Ca. Jersey cows easily develop peripartum hypocalcemia compared with other dairy cows. The early detection and treatment of hypocalcemia are particularly important for Jersey cows because delayed treatment can result in various complications. In this study, to establish a simple, noninvasive, on-site diagnosis of hypocalcemia in perinatal Jersey cows, we attempted to create an equation for estimating Ca using ECG waveforms. Overall, 112 Jersey cows 0–2 days postpartum were used. The ECG findings of these cows were measured using the base-apex lead for 30 s and the corrected ST interval (STc = ST peak interval/SS peak interval^0.5^) was calculated. Simultaneously, blood was collected from the tail vein, and the serum total Ca (tCa) and serum ionized Ca (iCa) were measured. Several items considered related to Ca were investigated. A strong positive correlation was observed between the tCa and iCa (*r* = 0.96). A positive correlation was observed between the tCa and STc^−1^ (*r* = 0.83). Furthermore, significant correlations were observed between skin temperature, calving number, vigor level, rumen movement, and auricle temperature (*p* < 0.05). Of these, multiple regression analysis was performed to calculate the tCa estimation formula with the STc and calving number (categorized into primipara, second parity, and third or more parity) as explanatory variables and the tCa as the objective variable (*r* = 0.85, *p* < 0.01). Of 15 postpartum Jersey cows, the estimation formula could mostly distinguish between cows with hypocalcemia, those with subclinical hypocalcemia, and normal cows. Blood Ca in peripartum Jersey cows can be noninvasively estimated using ECG variables and calving number.

## 1. Introduction

In animals, calcium is required for muscle contraction. Therefore, muscles relax as blood calcium concentration (Ca) decreases (1). This phenomenon is the same not only in skeletal and smooth muscle cells but also in cardiomyocytes (1), and fluctuations in Ca affect myocardial contraction and change the electrocardiogram (ECG) waveform.

In human medicine, a decrease in Ca prolongs the QT interval on ECGs (2, 3). Some authors have reported on the relationship between Ca and ECG waveforms in cattle. Littledike et al. (4) reported that the corrected QT interval (QTc) in cattle was proportional to the Ca in the low Ca region. Daniel et al. (5) reported that in cows, Ca were < 1.84 mmol/L when the QTc was >450 msec and > 2.09 mmol/L when the QTc was <400 msec. Furthermore, Matsuo et al. (6) reported a significant correlation between Ca and the QTc in cattle; however, estimating the Ca from ECG findings is difficult because the error is too large.

In contrast, Itoh et al. (7, 8) focused on the S and T waves, which show high peaks in cattle. The corrected ST interval (STc) was calculated by correcting the ST peak interval by the SS peak interval using Bazett’s equation (9) (STc [seconds] = ST peak interval [seconds]/SS peak interval [seconds]^0.5^). Then, in Holstein cows, it was clarified that there was a strong positive correlation between the inverse of the STc (STc^−1^) and Ca, and a method for estimating Ca using the STc^−1^ and calving number was reported. Although peripartum Jersey cattle are susceptible to develop hypocalcemia and have difficulties with recovery (10, 11), studies involving peripartum Jersey cattle have not been conducted.

In this study, we clarified the relationship between ECG waveforms and Ca in peripartum Jersey cows. Furthermore, to improve the accuracy of estimating Ca, we also clarified the relationship between Ca and other variables related to hypocalcemia [i.e., calving number, heart rate, vigor level, rumen movement, auricle temperature, and skin temperature (12)]. Using these variables, we created an equation for estimating the Ca and verified its effectiveness.

## 2. Materials and methods

### 2.1. Animals

This study was conducted using a total of 112 Jersey cows (parity: 3.7 ± 1.7) from 0 to 2 days postpartum on a dairy farm in Tokachi region, Hokkaido, Japan.

### 2.2. Sampling

ECG measurements were performed 3 h or more after milking. ECG was recorded for 30 s by the base-apex (A–B) lead using an ECG device (Harada Electronic Industry Ltd., Sapporo, Japan). Electrodes were positioned on the anterior border of the left shoulder blade (base: +) and 10 cm posterior to the left olecranon (apex: –). The ST peak interval and SS peak interval were extracted using the software Cattle ECG 2 (Hokkaido Research Organization Industrial Research Institute, Sapporo, Japan). Tail vein blood was collected in 5 mL plain tubes with rapid coagulation accelerator (Insepack II-D SMD750SQ-blue, Sekisui Medical, Tokyo, Japan). The collected venous blood was centrifuged at 3000 rpm for 15 min, and the serum was separated. The serum total Ca (tCa) was measured using a fully automated clinical chemistry analyzer (TBA-120FR, TOSHIBA Medical systems, Tokyo, Japan), and the serum ionized Ca (iCa) was measured using an ionized calcium analyzer (cobas b 221 system, Roche Diagnostics, Rotkreuz, Switzerland). All iCa values were adjusted to pH 7.4. Furthermore, vigor level, calving number, heart rate, rumen movement, auricle temperature, and skin temperature were recorded as variables related to hypocalcemia, other than ECG. The vigor level was categorized as “none” if there was obvious depression, including lying down, and as “normal” otherwise. Heart rate and rumen movement were determined through auscultation. Rumen movement was classified as “none” when audible motion was not detected during a 2 min auscultation and as “move” otherwise. Auricular and skin temperatures were confirmed through palpation. If they felt notably cold, they were classified as “cold” and as “warm” otherwise.

### 2.3. Confirmation of the correlation between the iCa and tCa

A correlation analysis between the iCa and tCa was performed. The correlation coefficient (*r*) and *p*-value (*p*) were evaluated using Pearson’s correlation coefficient test. While muscle contraction relies on iCa (1), clinical veterinarians often measure tCa to diagnose hypocalcemia. In this study, we opted to use tCa as the objective variable in the Ca estimation equation when a very high correlation was confirmed between the iCa and tCa.

### 2.4. Relationship between ECG variables and the tCa

The average of the STc was calculated from the ST peak intervals and SS peak intervals for 30 s. To clarify the relationship between the STc^−1^ and tCa, the correlation coefficient (*r*) and *p*-value (*p*) were obtained.

### 2.5. Correlation analysis of variables related to the tCa, other than ECG

We used the heart rate and calving number as continuous variables for analysis. Vigor level (0, none; 1, normal), rumen movement (0, none; 1, move), auricle temperature (0, cold; 1, warm), and skin temperature (0, cold; 1, warm) were used as categorical variables for analysis. Additionally, some previous reports on hypocalcemia in Jersey cows were analyzed by categorizing the calving number into primiparous and multiparous cows, or primiparous, second and third or more parity (13–15). Therefore, calving number 2 (0, primipara; 1, multipara) and calving number 3 (0, primipara; 1, second parity; 2, third or more parity) were also used for analysis. We performed correlation analysis for the tCa, STc^−1^, and aforementioned variables to obtain the correlation coefficient (*r*) and *p*-value (*p*) between each variable.

### 2.6. Creation of the formula for estimating the tCa

A multiple regression model was created using the tCa as the objective variable and STc and the variables related to the tCa, other than ECG, as the explanatory variables. The multiple regression equation for estimating the tCa, correlation coefficient (*r*), adjusted coefficient of determination (adjusted *R*^2^), and *p*-values (*p*) were calculated.

### 2.7. Verification of the estimation formula

To ascertain the credibility of the established multiple regression equation, we gathered ECG data and obtained tail vein blood from an additional 15 Jersey cows on the day following parturition. We then compared the estimated tCa using our prediction equation with the actual tCa. Clinical hypocalcemia was defined as tCa of <6.0 mg/dL (16, 17), and subclinical hypocalcemia was defined as tCa < 7.5 mg/dL (18).

### 2.8. Statistical analysis

All statistical analyses were performed using R (version 3.6.1). Correlation analyses between iCa and tCa, as well as between tCa and STc^−1^, were conducted using the Pearson’s correlation coefficient test subsequent to confirming normality through the Shapiro–Wilk test. For correlations involving tCa and variables excluding ECG, normality was verified via the Shapiro–Wilk test and then analyzed using Pearson’s correlation coefficient for normally distributed variables and Spearman’s rank correlation coefficient for other variables. The formula for estimating tCa was derived through multiple regression analysis. In the process of selecting explanatory variables, priority was accorded to those presenting objective numerical values and that could be incorporated into the estimation equation beforehand. Correlation coefficients (*r*) were considered to be very high at |*r*| > 0.9, high at |*r*| > 0.7, moderate at |*r*| > 0.50, and low at |*r*| > 0.3 (19). The goodness of fit of linear regression was considered acceptable at adjusted *R*^2^ > 0.5, according to a previous report (20, 21). Differences with *p*-values (*p*) < 0.05 were considered statistically significant.

### 2.9. Ethics statement

All protocols and procedures were approved by the Animal Care and Use Committee of Obihiro University of Agriculture and Veterinary Medicine (approval no. 20–89).

## 3. Results

**Figure 1:**
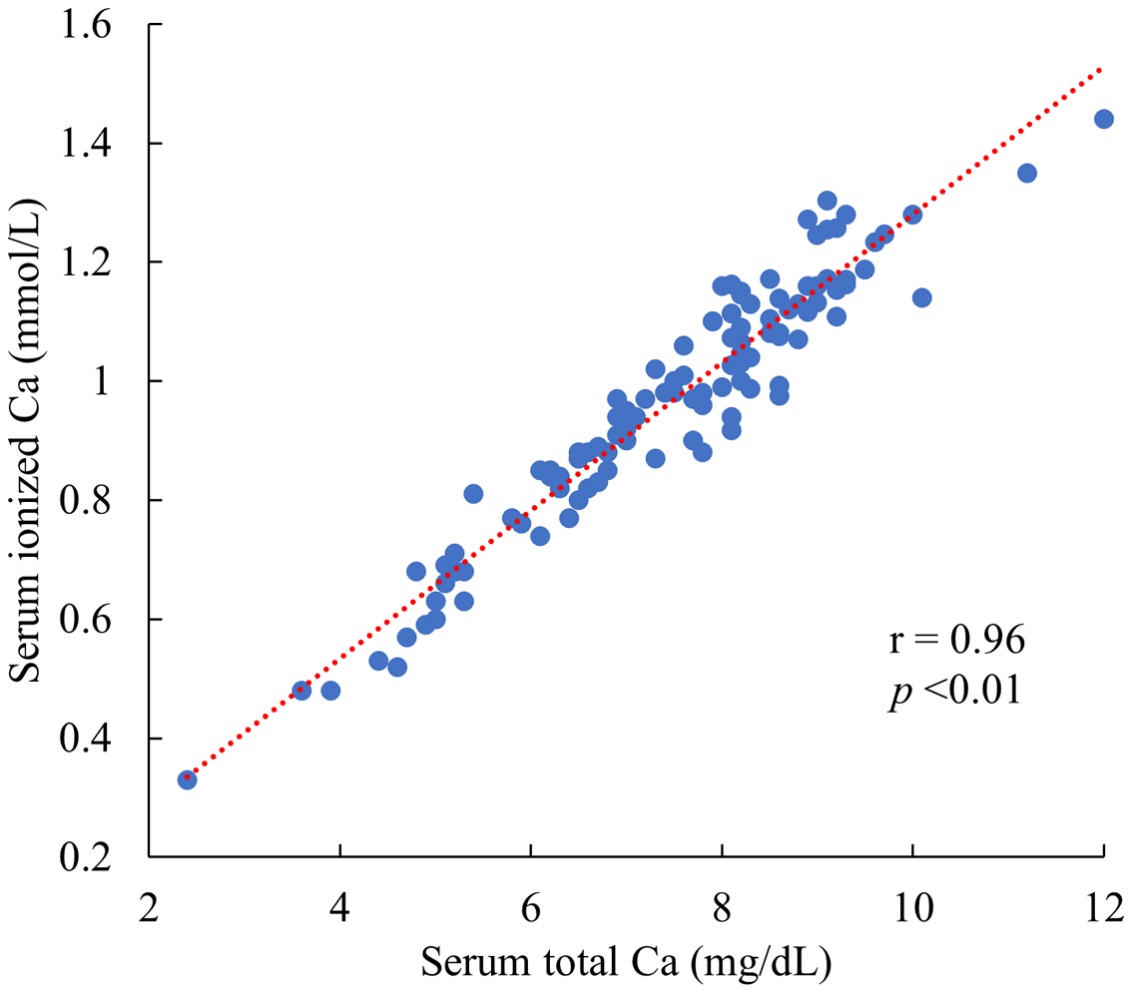
Relationship between serum ionized Ca and serum total Ca in 112 Jersey cows 0–2 days postpartum. (All data originated from Jersey cows located on a single dairy farm situated within the Tokachi region of Hokkaido, Japan).

A significant and very high correlation was confirmed between the iCa and tCa (*r* = 0.96, *p* < 0.01) in 112 cows (Figure 1). Therefore, an estimation formula was created using the tCa as an objective variable. Among these, 20 cows exhibited tCa below 6.0 mg/dL, and 31 cows fell within the range of tCa between 6.0 and 7.5 mg/dL.

**Figure 2:**
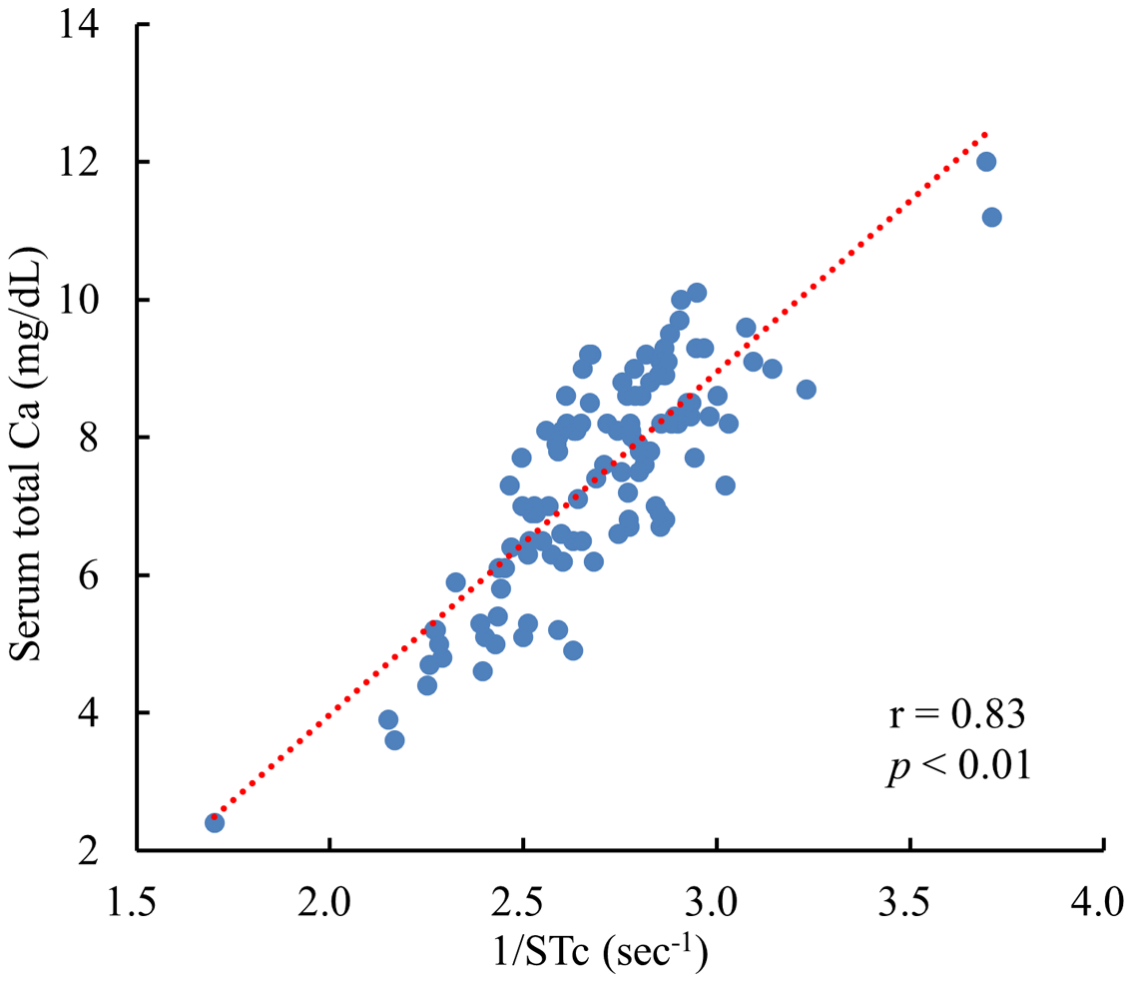
Relationship between the STc^−1^ and serum total Ca in 112 Jersey cows 0–2 days postpartum. STc^−1^: the inverse of the ST peak intervals corrected for the SS peak intervals (heart rate) by Bazett’s formula. (All data originated from Jersey cows located on a single dairy farm situated within the Tokachi region of Hokkaido, Japan).

A significant and strong positive correlation was observed between the tCa and STc^−1^ in Jersey cows (*r* = 0.83, *p* < 0.01) (Figure 2).

As variables related to the tCa other than the STc, a significant correlation was observed between skin temperature (*r* = 0.50, *p* < 0.01), calving number-3 (*r* = −0.30, *p* < 0.01), vigor level (*r* = 0.30, *p* < 0.05), rumen movement (*r* = 0.30, *p* < 0.05), auricle temperature (*r* = 0.29, *p* < 0.05), calving number (*r* = −0.25, *p* < 0.05), and calving number-2 (*r* = −0.23, *p* < 0.05).

In this study, we decided to select calving number-3, which is an objective numerical value and can be incorporated into the estimation formula in advance, among the variables with significant correlation. To derive a regression equation for estimating the tCa, multiple regression analysis was performed using the tCa as the objective variable and STc^−1^ and calving number-3 as explanatory variables. The following regression equation was obtained with *r* = 0.85, adjusted *R*^2^ = 0.72, and *p* < 0.01:


$$\begin{array}{c} Ca\left[\mathrm{mg}/\mathrm{dL}\right]=a\times 1/STc\left[\mathrm{seconds}\right]+b\times \mathrm{calving}\;\mathrm{number}\;\left[1\;\mathrm{or}\;2\;\mathrm{or}\;3*\right]+c\\ a:\;4.75\pm 0.31,\;b:\;-0.41\pm 0.13,\;c:\;-4.29\pm 0.97.\\ \mathrm{*: 1, primipara; 2, second parity; 3, third or more parity}\text{.} \end{array}$$

**Figure 3:**
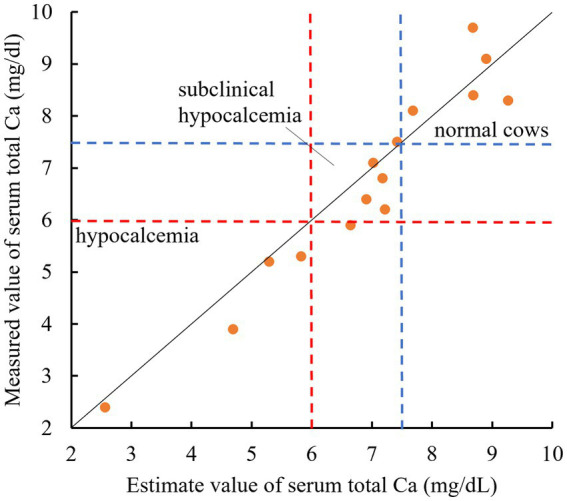
Relationship between the estimated and measured values of the serum total Ca in 15 parturient Jersey cows. The solid line shows the relationship between y = x. Values below the blue dotted line indicate serum total Ca < 7.5 mg/dL. Values below the red dotted line indicate serum total Ca < 6.0 mg/dL. (All data originated from Jersey cows located on a single dairy farm situated within the Tokachi region of Hokkaido, Japan).

The plot diagram of the measured and estimated tCa using 15 postpartum Jersey cows had *r* = 0.90 and *p* < 0.01. By employing thresholds of 6.0 mg/dL (assuming clinical hypocalcemia) and 7.5 mg/dL (assuming subclinical hypocalcemia), our estimation formula effectively differentiated between hypocalcemia and subclinical hypocalcemia in almost all cows (Figure 3). Of the four cows with estimated blood Ca of <6.0 mg/dL, three were lying down.

## 4. Discussion

Jersey cows have a higher occurrence rate of hypocalcemia than Holstein cows (10, 11). The rate of occurrence has been reported to be 5.4% in Holstein cows and 33.3% in Jersey cows (22). The main causes of this disparity are higher milk Ca and fewer intestinal 1,25(OH)_2_D_3_ receptors in Jersey cows than in Holstein cows (10). In this study, we used a total of 112 Jersey cows within 3 days postpartum that are susceptible hypocalcemia, and 48 showed hypocalcemia with tCa < 7.5 mg/dL.

Muscle contraction is dependent on the iCa (1). However, the tCa is more commonly measured than the iCa in many clinical fields (23–26). Although the iCa is usually considered to be approximately 45% of the tCa (27), because calcium in the blood is bound to albumin, the proportion of the iCa is higher in cows with hypoalbuminemia (28). Therefore, correcting blood albumin concentrations is necessary to evaluate hypocalcemia using the tCa (28). Although the blood albumin concentration was not shown in this study, it was confirmed that the iCa was very strongly correlated with the tCa; we considered that cows with hypoalbuminemia were excluded, and we created an estimation formula to calculate the tCa. However, developing a formula to estimate the iCa was also possible.

Electrolytes (Ca^++^, Na^+^ and K^+^) play a pivotal role in regulating the cyclic process of cardiac contraction and dilation. As these electrolytes move in and out of cardiomyocytes, they generate an electrical stimulus (potential difference) that propels heart contractions. Specifically, this electrical stimulation stems from Na^+^ influx into cardiomyocytes and is maintained by Ca^++^ influx (29). Any disturbance in electrolyte levels can impede electrical stimulation, consequently impacting cardiac function. In cases of hypocalcemia, the inflow of Ca^++^ into cardiomyocytes is delayed, leading to weakened cardiac contractions. Consequently, the ST interval on the ECG (QT interval in humans) experiences prolongation. Our previous report revealed an inverse relationship between tCa and STc in Holstein cows (7). This was also observed in Jersey cows. The regression analysis using the reciprocal of the STc showed a strong positive correlation between the tCa and STc^−1^ (*r* = 0.83). Using ECG in estimating the tCa was also effective in Jersey cows.

Vigor level, rumen movement, auricle temperature, and skin temperature in cows exhibited notable correlations with tCa and were identified as valuable explanatory variables for incorporation into the tCa estimation formula. These variables hold practical utility, as they can be easily tested on the farm. However, considering the practicality of tCa estimation using ECG in the field, we believe that attaching ECG to cows and monitoring their tCa from remote locations (e.g., home and clinic) would be valuable. Therefore, in this study, we selected the calving number, which can be incorporated into the estimation formula in advance, as the explanatory variable. When only the STc was used as an explanatory variable, the correlation coefficient was 0.83; however, when the calving number was added, the correlation coefficient increased to 0.85, improving the accuracy of the estimation formula. Recently, wearable sensors have also attracted attention in farm animals. In the future, it is expected that information on vigor, rumen movement, or skin temperature in cows will be available in real time using motion, acceleration, or temperature sensors. At that time, incorporating these variables into the estimation formula enables more accurate tCa estimation.

In this study, we investigated the following three types of variables for the calving number: “calving number,” which treats the number of parities as a continuous variable; “calving number-2,” which was classified into two categories (i.e., primiparous and multiparous); and “calving number-3,” which was classified into three categories (i.e., primipara, second parity, and third or more parity). As a result, “calving number-3,” which was classified into primipara, second parity, and third or more parity, had the highest correlation with the tCa, and this categorical variable was incorporated into the estimation formula. Hypocalcemia tends to occur in cows with a high calving number (30, 31); the formula for estimating Ca in Holstein cows used the calving number, which is a continuous variable (8). However, in this study, no relationship was observed between the calving number and Ca in cows with 3 or more parity. Given Jersey cows’ heightened susceptibility to hypocalcemia compared to Holstein cows (10, 11), it is possible that these animals might have encountered severe hypocalcemia by their second and third parity, potentially leading to culling. As a result, it is possible that only individuals who were less likely to develop hypocalcemia had repeated pregnancies and parturition. Verifying whether this phenomenon occurs in this farm only or whether it is generally observed is necessary.

A comparison of tCa estimates and tCa measurements using 15 parturient cows confirmed that the present formula derived from the STc and calving number can noninvasively predict Ca (i.e., hypocalcemia, subclinical hypocalcemia, or normal) in Jersey cows. Detecting subclinical hypocalcemia without blood sampling is important in managing hypocalcemia. ECG measurement in cows using AB leads is very simple. By incorporating an algorithm to extract the STc interval into the ECG, hypocalcemia in Jersey cows can be instantly determined without blood sampling. These are particularly useful in farms with inexperienced staff who cannot determine hypocalcemia from clinical signs. Furthermore, in the future, continuous 24 h ECG measurements in peripartum cows will enable real-time monitoring of hypocalcemia.

Only a few farms breed Jersey cows in Japan, and the farm used in this study was the only farm that breeds several Jersey cows in our city. In the future, we plan to increase the number of surveyed animals to improve the accuracy of the Ca estimation formula; furthermore, we should also increase the number of farms.

## Data availability statement

The raw data supporting the conclusions of this article will be made available by the authors, without undue reservation.

## Ethics statement

The animal studies were approved by Animal Care and Use Committee of Obihiro University of Agriculture and Veterinary Medicine (approval no. 20–89). The studies were conducted in accordance with the local legislation and institutional requirements. Written informed consent was obtained from the owners for the participation of their animals in this study.

## Author contributions

MI and SK contributed to the conception and design of the study. IN organized the database. YC performed the statistical analysis and wrote the first draft of the manuscript. YC and MI wrote sections of the manuscript. All authors contributed to the article and approved the submitted version.

## References

[1] Klein BG Cunningham JG. The physiology of muscle In: Cunningham JG Klein BG, editors. Textbook of veterinary physiology. 4th ed. Amsterdam: Elsevier (2007). 81–90.

[2] Bronsky D Dubin A Waldstein SS Kushner DS. Calcium and the electrocardiogram: I. the electrocardiographic manifestations of hypoparathyroidism. Am J Cardiol. (1961) 7:823–32. doi: 10.1016/0002-9149(61)90401-5

[3] Wagner GS Lim TH. Interpretation of the normal electrocardiogram In: Wagner GS, editor. Marriott's practical electrocardiography. 11th ed. Philadelphia, PA: Lippincott Williams & Wilkins (2007). 44–71.

[4] Littledike ET Glazier D Cook HM. Electrocardiographic changes after induced hypercalcemia and hypocalcemia in cattle: reversal of the induced arrhythmia with atropine. Am J Vet Res. (1976) 37:383–8. PMID: 1267235

[5] Daniel RCW Moodie EW. Relationship between plasma calcium and QT interval of electrocardiogram in dairy cows. J Dairy Sci. (1979) 62:1014–8. doi: 10.3168/jds.S0022-0302(79)83365-2, PMID: 500894

[6] Matsuo N Takahashi K Kurosawa T Sonoda M. Changes in electrocardiogram by serum calcium concentration in cattle [Japanese]. J Jpn Vet Med Assoc. (1987) 40:408–12. doi: 10.12935/jvma1951.40.408

[7] Itoh M Sakurai Y Nakajima Y Kawamoto S. Relationship between blood calcium level and ST peak interval of electrocardiographic variables in peripartum Holstein cows. J Vet Med Sci. (2015) 77:1655–7. doi: 10.1292/jvms.15-0205, PMID: 26118411 PMC4710724

[8] Itoh M Nakajima Y Kuwano K Maeda D Sakurai Y Matsui Y. Improving the accuracy of estimating blood calcium concentration in Holstein cows using electrocardiographic variables. J Vet Med Sci. (2022) 84:193–8. doi: 10.1292/jvms.21-0320, PMID: 34897186 PMC8920712

[9] Bazett HC. An analysis of the time-relations of electrocardiogram. Heart. (1920) 7:353–70.

[10] Goff JP. Acute hypocalcemia in dairy cows In: Smith BP, editor. Large animal internal medicine. 5th ed. Amsterdam: Elsevier (2014). 1259–62.

[11] Oezel GR Goff JP. Milk fever (parturient paresis) in cows, ewes, and doe goats In: Anderson DE Rings DM, editors. Current veterinary therapy food animal practice. 5th ed. Philadelphia, PA: Saunders (2009). 130–4.

[12] Radostits OM Gay CC Hinchciff KW Constable PD. Parturient paresis (milk fever) In: Veterinary medicine; a textbook of the disease of cattle, horses, sheep, pigs, and goats. 10th ed. Amsterdam: Elsevier (2007). 1626–44.

[13] Taylor MS Knowlton KF McGilliard ML Seymour WM Herbein JH. Blood mineral, hormone, and osteocalcin responses of multiparous Jersey cows to an Oral dose of 25-Hydroxyvitamin D3 or vitamin D3 before parturition. J Dairy Sci. (2008) 91:2408–16. doi: 10.3168/jds.2007-0750 18487663

[14] Reitsma LM Batchelder TA Davis EM Machado VS Neves RC Ballou MA. Effects of oral calcium bolus supplementation on intracellular polymorphonuclear leukocyte calcium levels and functionality in primiparous and multiparous dairy cows. J Dairy Sci. (2020) 103:11876–88. doi: 10.3168/jds.2020-18835, PMID: 33069401

[15] Menta PR Fernandes L Poit D Celestino ML Machado VS Ballou MA. Association of blood calcium concentration in the first 3 days after parturition and energy balance metabolites at day 3 in milk with disease and production outcomes in multiparous Jersey cows. J Dairy Sci. (2021) 104:5854–66. doi: 10.3168/jds.2020-19189 33612230

[16] McArt JAA Neves RC. Methods of measurement and implications of abnormal calcium concentrations in fresh dairy cows. AABP Proc. (2017) 50:81–4. doi: 10.21423/aabppro20173283

[17] Jeong JK Kang HG Kim IH. Associations between serum calcium concentration and postpartum health and reproductive performance in dairy cows. Anim Reprod Sci. (2018) 196:184–92. doi: 10.1016/j.anireprosci.2018.08.006, PMID: 30120012

[18] Zinicola M Korzec H Teixeira AGV Ganda EK Bringhenti L Tomazi ACCH. Effects of pegbovigrastim administration on periparturient diseases, milk production, and reproductive performance of Holstein cows. J Dairy Sci. (2018) 101:11199–217. doi: 10.3168/jds.2018-14869, PMID: 30316593

[19] Asuero AG Sayago A Gonzalez AG. The correlation coefficient: an overview. Crit Rev Anal Chem. (2006) 36:41–59. doi: 10.1080/10408340500526766

[20] Billaut F Buchheit M. Repeated-sprint performance and vastus lateralis oxygenation: effect of limited O_2_ availability. Scand J Med Sci Sports. (2013) 23:e185–93. doi: 10.1111/sms.12052, PMID: 23362832

[21] Moriasi DN Arnold JG Van Liew MW Bingner RL Harmel RD Veith TL. Model evaluation guidelines for systematic quantification of accuracy in watershed simulations. Trans ASABE. (2007) 50:885–900. doi: 10.13031/2013.23153

[22] Hibbs JW Krauss WE Monroe CF Sutton TS. Studies on milk fever in dairy cows I. the possible role in vitamin D in milk fever. J Dairy Sci. (1946) 29:617–23. doi: 10.3168/jds.S0022-0302(46)92514-3

[23] Barraclough RAC Shaw DJ Thorup VM Haskell MJ Lee W Macrae AI. The behavior of dairy cattle in the transition period: effects of blood calcium status. J Dairy Sci. (2020) 103:10604–13. doi: 10.3168/jds.2020-18238, PMID: 32896414

[24] Caixeta LS Ospina PA Capel MB Nyda DV. Association between subclinical hypocalcemia in the first 3 days of lactation and reproductive performance of dairy cows. Theriogenology. (2017) 94:1–7. doi: 10.1016/j.theriogenology.2017.01.039, PMID: 28407850

[25] Venjakob P Borchardt S Heuwieser W. Hypocalcemia – cow – level prevalence and preventive strategies in German dairy herds. J Dairy Sci. (2017) 100:9258–66. doi: 10.3168/jds.2016-12494, PMID: 28865859

[26] Blanc CD Van der List M Aly SS Rossow HA. Blood calcium dynamics after prophylactic treatment of subclinical hypocalcemia with oral or intravenous calcium. J Dairy Sci. (2014) 97:6901–6. doi: 10.3168/jds.2014-7927 25200776

[27] Ott D Schrapers KT Aschenbach JR. Changes in the relationship between ionized and total calcium in clinically healthy dairy cows in the period around calving. Animals. (2021) 11:1036. doi: 10.3390/ani11041036, PMID: 33917559 PMC8067466

[28] Seifi HA Mohri M Ehsani A Hosseini H Chamsaz M. Interpretation of bovine serum total calcium: effects of adjustment for albumin and total protein. Comp Clin Pathol. (2005) 14:155–9. doi: 10.1007/s00580-005-0582-2

[29] Grant AO. Cardiac ion channels. Circ Arrhythm Electrophysiol. (2009) 2:185–94. doi: 10.1161/CIRCEP.108.789081, PMID: 19808464

[30] Miltenburg CL Duffield TF Bienzle D Scholtz EL LeBlanc SJ. Randomized clinical trial of a calcium supplement for improvement of health in dairy cows in early lactation. J Dairy Sci. (2016) 99:6550–62. doi: 10.3168/jds.2016-10961, PMID: 27265174

[31] Curtis CR Erb HN Sniffen CJ Smith RD. Epidemiology of parturient paresis: predisposing factors with emphasis on dry cow deeding and management. J Dairy Sci. (1984) 67:817–25. doi: 10.3168/jds.S0022-0302(84)81372-7, PMID: 6725726

